# Incidence and Risk Assessment for Atrial Fibrillation at 5 Years: Hypertensive Diabetic Cohort

**DOI:** 10.3390/ijerph17103491

**Published:** 2020-05-16

**Authors:** Eulalia Muria-Subirats, Josep Lluis Clua-Espuny, Juan Ballesta-Ors, Blanca Lorman-Carbo, Iñigo Lechuga-Duran, Jose Fernández-Saez, Roger Pla-Farnos, on behalf members of AFRICAT Group

**Affiliations:** 1 Department of Primary Care, Catalonian Health Institute, Institute for Research in Primary Health Care Jordi Gol i Gurina (IDIAPJGol) Rovira i Virgili University, 43500 Tortosa, Spain; jlclua@telefonica.net; 2 Department of Primary Care, UUDD Terres de l’Ebre-Tortosa, Catalonian Health Institute, 43500 Tortosa, Spain; juan.ballesta.ors@gmail.com (J.B.-O.); blancalormancarbo@gmail.com (B.L.-C.); 3 Department of Cardiology, Catalonian Health Institute, Hospital Verge de la Cinta, Institut de Recerca Sanitària Pere Virgili (IISPV), 43500 Tortosa, Spain; ilechuga.ebre.ics@gencat.cat; 4 Unitat de Suport a la Recerca Terres de l’Ebre, Fundació Institut Universitari per a la recerca a l’Atenció Primària de Salut Jordi Gol i Gurina (IDIAPJGol), 43500 Tortosa, Spain; jfernandez@idiapjgol.info; 5 Unidat de Recerca, Gerència Territorial Terres de l´Ebre, Institut Catalá de la Salut, 43500 Tortosa, Spain; 6 Facultat de Enfermería, Campus Terres de l´Ebre, Universitat Rovira i Virgili, 43500 Tortosa, Spain; 7 Primary Care Research Group, Department of Medicine and Surgery, Rovira I Virgili University, 43003 Tarragona, Spain; rpla@comt.es; 8 AFRICAT Research Group, Institute for Research in Primary Health Care Jordi Gol i Gurina (IDIAPJGol), Gran Via de les Corts Catalanes, 587, 08007 Barcelona, Spain

**Keywords:** risk assessment, hypertensive, diabetes, Atrial Fibrillation, stroke, Chronic diseases, cohort study

## Abstract

(1) Background: The link between diabetes and hypertension is mutual and reciprocal, increasing the risks for the development of atrial fibrillation (AF). The main objective was to develop a prediction model for AF in a population with both diabetes and hypertension at five years of follow-up. (2) Methods: A multicenter and community-based cohort study was undertaken of 8237 hypertensive diabetic patients without AF between 1 January 2103 and 31 December 2017. Multivariate Cox proportional-hazards regression models were used to identify predictors AF and to stratify risk scores by quartiles. (3) Results: AF incidence was 10.5/1000 people/years (95% confidence interval (CI) 9.5–11.5), higher in men. The independent prognostic factors identified: age (hazard ratio (HR) 1.07 95% CI 1.05–1.09, *p* < 0.001), weight (HR 1.03 95% CI 1.02–1.04, *p* < 0.001), CHA_2_DS_2_VASc score (HR 1.57 95% CI 1.16–2.13, *p* = 0.003) and female gender (HR 0.55 95% CI 0.37–0.82, *p* = 0.004). Q4 (highest-risk group for AF) had the highest AF incidence, stroke and mortality, and the smallest number needed to screen to detect one case of AF. (4) Conclusions: Risk-based screening for AF should be used in high cardiovascular risk patients as the hypertensive diabetics, for treatment of modifiable cardiovascular risk, and monitoring AF detection.

## 1. Introduction

The link between diabetes and hypertension is mutual and reciprocal, increasing the risks for the development of atrial fibrillation (AF). The incidence of diabetes mellitus and hypertension is assuming prevailing proportions. In Europe, the prevalence of AF is estimated at 10% for people aged ≥60 years, with 20.1% unknown. The prevalence of AF is expected to increase 2.5 times in the next 50 years [1,2] and approximately one-third of ischemic strokes are associated with the presence of AF [3]. The presence of diabetes and hypertension in patients with AF is associated with increased symptom burden and increased cardiovascular and cerebrovascular mortality [1,4], lower quality of life, higher mortality and higher hospitalization rates [5]. Many different cardiovascular risk factors related to the incidence of AF have been identified, but on average, patients with hypertension and diabetes mellitus [6,7,8,9] are considered to be at high or very high cardiovascular risk, although their individual residual risk ranges from low to very high. This underlines the need for cardiovascular risk stratification and calls for specific risk-prediction tools for these patients [10,11].

Despite the progress in AF diagnosis and management [4], prevention of stroke remains the cornerstone, because AF may remain undiagnosed until an ischemic stroke occurs. A major gap in stroke prevention is the prevalence of undetected AF in the community. Furthermore, this arrhythmia is associated with an increased risk of cognitive impairment, heart failure, sudden death and cardiovascular morbidity [5]. Consequently, it is a serious public health problem that generates significant health costs [3]. For all this, numerous organizations are advising the opportunistic detection of AF [12] as a strategy for its early diagnosis [13]. However, their results are disparate in effectiveness and the evidence suggests a need for the development of AF incidence risk scores and the use of new monitoring technologies in high-risk subjects [14,15,16,17,18] such as hypertensive diabetics.

The main objective of this study was to develop a multivariate prediction model for AF in a population with both diabetes and hypertension at five years of follow up.

## 2. Materials and Methods

### 2.1. Study Design

This was a multicenter and retrospective community-based study in general practice, including a cohort of 8237 patients with both diabetes (DM) and hypertension (HTA) without a known diagnosis of AF between 01/01/2013 and 31/12/2017. The follow-up was carried out until the patient’s death or until the end of the study. The reporting is compliant with the STROBE checklists standards.

### 2.2. Setting and Study Population

The study was conducted by 11 primary health care teams (Terres de l’Ebre) and a referral hospital (Figure S1), all managed by the Catalan Institute of Health (ICS). The data were obtained in an anonymized fashion from a computerized database retrospectively provided by the Information and Communication Technology Department from the Minimum Basic Data Set at hospital discharge register (CMBD-HA) using specific International Classification of Diseases (ICD–10) code prefixes for HTA (I10–I15), DM (E11–E14), AF (I48) and ischemic stroke (I60–I69) to principal investigators in a fully de-identified format; and supervised and analyzed according to the General Data Protection Regulation of Europe from 01/02/2017.

The inclusion criteria were people ≥18 years old with both HTA and DM diagnosis registered in their clinical record without known AF before the inclusion, residence in the study territory, and active medical history in any of the participating primary care centers. The exclusion criteria are the absence of registration in the medical history or the failure to meet the inclusion criteria. Currently, 98.2% of the population surveyed in the area had an active medical history.

### 2.3. Research Ethics

The study is part of the AFRICAT project [research Marató TV3: Grant number: 201528-30-31-3. ClinicalTrials.gov ID: NCT03188484] about a sequential screening program in a high-risk population of AF by integrating clinical, electrocardiographic and biological information.

The exploitation of the data was carried out anonymously according to the data protection regulations for medical research of the Declaration of Helsinki.

For this type of study, formal consent is not required and the requirement for the informed consent of patients was waived prior to the inclusion of their medical data in this study.

The research protocol was reviewed and approved by the ethics committee of the Institut Universitari d’Investigació en Atenció Primària Jordi Gol with code number 20-051P.

### 2.4. Study Variables

The primary outcome was the ID (incidence density) of AF and, ID stroke, all causes mortality and number-needed-to-screen for one AF case (NNS) as secondary outcomes.

All cases of new AF were confirmed by a referent cardiologist and/or supported with information from medical charts or other assessments. A stroke during the study period was considered to be present if the medical charts included a stroke diagnosis and it was supported with information from medical charts. Two investigators independently verified clinical diagnosis for AF and stroke (the principal investigator and a local investigator in charge of checking data of cases assigned to primary care center). In case of non-coincidence, they were removed from the analysis.

The following were included as independent variables (Tables S1 and S2): sociodemographic, clinical, cardiovascular comorbidities, type and number of active prescriptions and all included variables in the CHA_2_DS_2_VASc score. The heart rate was calculated as the average of all registered measures along the study or in the last year before the new AF diagnosis. The electronical medical records database of the ICS includes data from primary care, specialty clinics and hospitalizations along the year previous from the study begin

The datasets generated during and/or analyzed during the current study are available in the public repository.

### 2.5. Statistical Analysis

Descriptive statistics were used to summarize the baseline characteristics (24 variables) (Table 1). The ID rate (per 1000/people/years) of new AF and stroke, odds ratios, mortality rates and NNS, were calculated by population subgroups and risk stratum.

Multivariate Cox proportional-hazards regression models were used to identify predictors, confounding and predictive factors of each event. All variables which have a significant *p*-value ≤ 0.05 at basal differences between groups with/without AF were introduced in the multivariate model considering its clinical significance regardless of its removal by the procedure. A base model including simultaneously age, gender, weight, CHA_2_DS_2_VASc score, heart rate, diastolic blood pressure, antidiabetic and antihypertensive medication was fitted. All models included these variables and the others were separately added to the base model. A sex interaction was used for these covariates (14 variables) and AF (Table S3).

**Figure 1:**
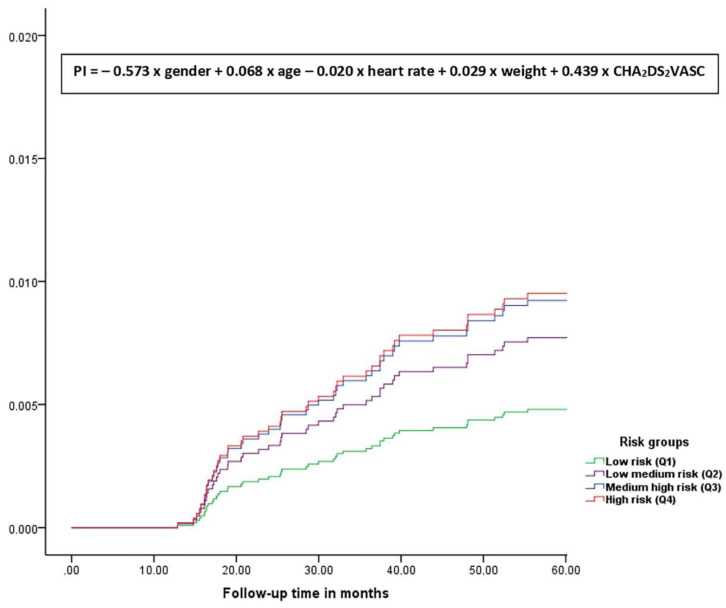
Function for predicting AF risk at 5 years.

With the Cox regression coefficients, a mathematical formula was constructed to measure the risk for a new AF: PI = − 0.573 × gender + 0.068 × age − 0.020 × heart rate + 0.029 × weight + 0.439 × CHA_2_DS_2_VASc (Figure 1); this formula was called the prognostic index (PI). This PI was a prognostic value associated to AF incidence risk at 5 years: probability of AF per 1000/people/years (Figure 1). The formula was applied to all hypertensive diabetic people managed in the study county (Terres de l’Ebre, Catalonia, Spain). After obtaining a PI value for each included case (8237 cases), it was stratified by quartiles (from Q1 the lowest risk for new AF to Q4 the highest risk for new AF), AF incidence intervals, mortality for all causes and stroke incidence.

We plotted the receiver operating characteristic (ROC) curves and computed the area under the curve (AUC) to assess the prediction power of the models. Calibration was assessed by the shrinkage coefficient and also calculated the D-statistic (a measure of discrimination where higher values indicate better discrimination). To ensure internal validation, we performed a 10-fold cross-validated multivariate regularized logistic regression to predict new AF based on all other variables significant of the baseline characteristics. Tests were performed with random samples selected with SPSS with 20% of the total cases. Once this was verified, the bootstrap method was used.

Time-to-event analysis was performed using non-parametric methods like the Kaplan–Meier and Log-Rank tests. The analysis was performed with IBM SPSS Statistics 10.0, the Excel 2010 and Stata 14.

## 3. Results

### 3.1. Baseline Characteristics

We included 8237 cases (women 49%) in the baseline study, with an average age of 78.01 ± 11.9 years (women 80.2 ± 11.6 vs. men 75.9 ± 11.8, *p* = 0.017) and average follow-up time of 4.9 ± 0.7 years. The baseline characteristics are shown in Table 1 The stroke prevalence was higher in patients with HTA/DM and AF (15.3%) than in patients with HTA/DM without AF (0.01%). Females with AF were older and had more vascular comorbidities, especially strokes (*p* < 0.001). The percentage of females with AF (41.6%) was lower than that of males (*p* = 0.001), but their average age (*p* < 0.001) was higher.

The overall AF incidence was 10.5/1000 people/years (95% confidence interval (CI) 9.5–11.5), higher in men than in women (12.21 95% CI 10.74–13.82 vs. 9.04 95% CI 7.76–10.47, *p* < 0.001) with an odds ratio (OR) of 1.35 (95% CI 1.10–1.65, *p* = 0.002). In relation to age, the AF ID rate was higher in men in all age groups <90 years with a men/women OR of 1.45 (95% CI 1.16–1.82, *p* = 0.001); there were differences according to the CHA_2_DS_2_VASc score (Table 2). A CHA_2_DS_2_VASc score ≥4 was associated with and OR of 1.94 (95% CI 0.29–3.59) for a new AF. The incidence density of a new AF increased progressively up to a score 5 in men and up to 7 in women.

The overall mortality was 26.7% [55.5/1000 people/years (95% CI 53.21–57.88)] without differences between those with/without AF (*p* > 0.05).

### 3.2. Independent Prognostic Factors for New AF

The adjusted multivariate model (Table 3) identified the following independent prognostic factors associated to risk for new AF: CHA_2_DS_2_VASc (HR 1.57 95% CI 1.16–2.13, *p* = 0.003), age (hazard ratio (HR) 1.07 95% CI 1.05–1.09, *p* < 0.001), weight (HR 1.03 95% CI 1.02–1.04, *p* < 0.001), heart rate (HR 0.98 95% CI 0.97–0.99, *p* = 0.001) and female gender (HR 0.55 95% CI 0.37–0.82, *p* = 0.004).

Heart rate showed an indirectly proportional relationship with the AF ID rate. The cut off [< 60 vs. > 90] was the point for significant differences in the incidence density of AF: the highest heart rate, the lowest AF incidence: OR 3.58 [95% CI 0.71–6.62] in patients with average heart rate < 60 vs. > 90 bpm; 1.67 [95% CI 1.34–1.87] in <60 vs. 60–90 bpm; and 0.47 [95% CI 0.16–1.13] in > 90 vs. 60–90 bpm. The incidence of AF was not modified by the use of beta-blockers (*p* = 0.433). In a similar way, those with a weight ≥90 kg had a higher AF incidence vs. those with <90 kg [14.1 (95% CI 11.91–16.75) vs. 9.8 (95% CI 8.7–11.0), *p* = 0.003].

### 3.3. Risk Score for New Atrial Fibrillation (AF) (Prognostic Index, PI)

The total risk score ranges from a first quartile (≤6.88) as lowest AF risk up to fourth quartile (>8.39) as highest AF risk **(Figure 1).** The average was 7.55 and 7.71 the median. After calculating the individual score, the subjects were stratified in quartiles according their score. The interquartile range (IQR) was 1.51. The estimated prediction of developing AF at 5 years of follow-up according to the individual risk score was:

1. Q1 (PI ≤ 6.88). Lowest risk group for AF: ID rate 2.95/1000 people/years (95% CI 1.69–4.80).
2. Q2 (PI 6.89 ≤ 7.71). Median-low risk group for AF: ID rate 8.85/1000 people/years (95% CI 6.54–11.70).
3. Q3 (PI 7.72 ≤ 8.39). Median-high risk group for AF: ID rate 15.70/1000 people/years (95% CI 12.47–19.52).
4. Q4 (PI > 8.39). Highest risk group for AF: ID rate 22.45/1000 people/years (95% CI 18.41–27.10).

The highest-risk group for AF (Q4) was characterized by a higher age (85.95 ± 6.03, *p* < 0.001), proportion of women (85.2%, *p* < 0.001), AF ID rate (22.5/1000 people/years), stroke ID rate (3.5/1000 people/years), total mortality (22.7%, *p* < 0.001) and lowest NNS (6). The AF ID rate was different by sex (women 19.7/1000 people/years vs. men 37.6/1000 people/years, *p* < 0.001) (Table 4). The internal validity of the model showed an AUC of 0.670 (95% CI 0.63–0.70, *p* < 0.001) with a predictive positive value increasing from Q1 (6.2%) to Q4 (42.5%) but with a similar predictive negative value (73.5% vs. 77.3%).

Results showed 71.05% of the new strokes occurred among those patients with new AF in the follow-up period of the whole cohort, especially among men included in the Q4 group (4.0/1000 people/years) (Table 4).

## 4. Discussion

### 4.1. Main Findings

The proposed model provides clear added value in the identification of those individuals with the highest risk of suffering AF in asymptomatic population as well as about the differences by sex in the distribution of risk of suffering AF and stroke. This may allow identification of a population with specific high-risk features among those patients at higher risk of developing AF, who may subsequently benefit from intensive screening strategies compared to those at lower risk. Given that the scenario was not the performance of a test on a patient but the population screening, the positive predictive value (PPV) will depend on the probability that the individual ends up having an AF. In this way, the likelihood ratio would be more interesting to determine whether the probability of AF diagnosis increases. Previous studies in the general population reported 147 cases (NNS) required for a new AF diagnosis using the opportunistic detection procedure [19] and 10/1000 people/years as estimated incidence [2]. If they are compared with the NNS (n = 9) and new AF incidence (22.5/1000 people/years) at Q4 risk quartile from current study, there would be an estimated likelihood enough to use in the hypertensive diabetic population.

The group with highest risk of AF, also had the highest incidence of stroke and mortality, and the smallest NNS to detect one AF case compared to the usual results of population screening [20,21]. The highlight of the study was that the 86% of new AF cases among women occurred in the highest risk group (Q4), and 71% of new strokes occurred among those with new AF. The screening for AF to improve the detection results of new AF cases and intensive modification of risk factors in these target groups should be considered [22,23]. In spite of the total incidence of AF in this cohort of hypertensive diabetic patients not differing from that of the general population [2,24], the NNS together with the two risk factors would be sufficient for targeting screening. In spite of the hypertension and diabetes being recognized as risk factors for cardiovascular diseases, a lower prevalence rate of peripheral vascular disease and congestive heart failure than previous reports [25] was identified. The list of factors associated with their incidence is increasing [6,18,25,26,27] and their high prevalence as associated comorbidity may play more of a risk role for the incidence of other cardiovascular events such as stroke than being a direct etiologic agent [28]. In any case, the current recommendations [12,26] are just focused on their appropriate control as risk factors and the early approach to AF as an important risk factor for other cardiovascular diseases, especially stroke [1,2,3].

### 4.2. Interpretation of the Study Results

Among the risk factors associated with new AF, the heart rate has been associated with the progression of AF [29] independent of rhythm, but not with new AF. However, in patients with heart failure an elevated heart rate was an independent predictor of adverse cardiovascular outcomes in patients in sinus rhythm, even after adjustment for NT-proBNP but there was no relationship between heart rate and outcomes in AF [30]. Age is one factor that showed a stronger association in women than in men, which could be explained by the greater longevity of women. Older age, especially of women at risk, may condition the prognostic value of the variables included in the different scales. Although, women and men share risk factors for AF, the gender differences for prior cardiovascular disease prevalence are well known [31], but the development of AF in women would require long-term follow-up by differential associations of classical risk factors. The link between obesity and AF has been supported by data from several large cohort studies [32] and the incidence of AF in diabetic subjects was double that of non-diabetic subjects after correction for multiple risk factors, and appears as a potential target for intervention preferable [33] to optimize chances of durable rhythm control.

The women/men ischemic stroke risk ratio varied with risk level and CHA_2_DS_2_VASc score [34,35]. In men, 80% of new AF occurred in the Q1–Q3 group, and the stroke incidence was significantly higher in Q4. However, 85.9% of new AF cases in women occurred at the Q4 level, while there were no differences in stroke incidence among the risk levels. These differences could be explained by the fact that, with women’s gender being protective, the incidence of AF would be conditioned by the presence of a greater number of vascular comorbidities and higher thrombotic risk. The CHA_2_DS_2_VASc score has been suggested to detect subjects with a risk of new AF and a stroke risk in subjects without AF [12,18,21]. Our results also support CHA_2_DS_2_VASc as a useful assessment to identify subjects with a high risk of AF; however, their relationship with strokes would differ by gender. These results suggest using the Q4 level in women and Q3/Q4 levels in men as targets in the screening activities.

Regarding the usefulness of risk scales [31,36,37,38], the inclusion of new prognostic variables, their accessibility from primary care, their prognostic time, and their cost-effectiveness [6] in relation to the main objective should be considered: AF detection vs. prevention ictus. Our study did not use echocardiographic data [23,39] but shows specificities by gender.

In stroke registries, at least one-third of patients with ischemic stroke had either previously identified [3] or newly detected AF at the time of stroke. Therefore, screening of the AF group is very important for stroke prevention. The association with AF is even higher if prolonged post-stroke external or implanted monitoring is performed [40]. The results confirm the concentration of most strokes and their highest incidence in the highest-risk group among men. Clearly, the risk-based screening for the evaluation of AF could help decide not only the best target population, but also which methods work best in initiating the early introduction of oral anticoagulant therapy.

### 4.3. Strengths and Limitations

The proposed model provides a clear added value in the identification of those individuals with the highest risk of suffering AF and, therefore, monitoring them more effectively to prevent stroke in the real world practice of primary care. All hypertensive patients with diabetes should be screened to identify their risk for AF. The model is based on data likely to be available to the primary physician at any time point without the need for further laboratory testing or imaging studies. Therefore, given that the scenario was not the performance of a test on a patient but population screening, future research is needed by comparing the pre-and post-test probabilities to determine whether the probability of AF diagnosis increases; and directed towards the cost-effectiveness analysis of a protocol that includes the systematic identification of target people at high risk for AF, the modification of risk factors, and the use of echocardiography criteria and biomarkers to define those subjects upon whom specific monitoring should be carried out (to be defined), with the main objective of reducing the incidence of their comorbidities, especially stroke.

Among the limitations, the study was not randomized; potentially, the AF risk estimation may overestimate cardiovascular risk in elderly individuals; and, given that some variables are item present also in the CHA_2_DS_2_VASc, a collinearity interpretation should be performed for highly correlated variables. Eventually, the results need an external validation.

## 5. Conclusions

This is a study of clinical records, looking at the incidence of AF and stroke in people with hypertension (HTA) and diabetes (DM). The highest-risk group was characterized by a higher AF ID rate (22.5/1000 people/years), stroke ID rate (3.5/1000 people/years), total mortality (22.7%, *p* < 0.001), and lowest NNS. There was a difference in stroke rate for the AF (15.3%) vs. non-AF (0.01%) patients within 5 years. Risk-based screening for AF should be used as a tool to define the target population and to improve the effectiveness of AF screening methods in hypertensive diabetics, for the treatment of modifiable cardiovascular risk, and monitoring new methodology for AF detection.

## 6. Patents

There is one patent resulting from this work that is managed by IDIAP Institut de Recerca en Atenció Primària Jordi Gol i Gurina.

## Figures and Tables

**Table 1:** Baseline characteristics with/without atrial fibrillation (AF).

	No AF ^a^	AF ^a^	*p* ^b^
***N* (%)**	7809 (94.8%)	428 (5.2%)	<0.001
**Average time tracking (years) ($\bar{x}$ ± ds ^c^)**	4.89 ± 0.71	4.61 ± 1.08	<0.001
**Age (years) ($\bar{x}$ ± ds)**	77.83 ± 12.01	81.36 ± 8.59	<0.001
**Women n (%)**	3856 (49.4%)	178 (41.6%)	0.001
**Weight (kg) ($\bar{x}$ ± ds)**	81.04 ± 16.18	84.15 ± 16.98	0.005
**BMI ^d^ (kg/m^2^) ($\bar{x}$ ± ds)**	31.10 ± 5.62	32.12 ± 5.77	0.010
**Systolic blood pressure (mmHg) ($\bar{x}$ ± ds)**	138.87 ± 14.32	140.02 ± 14.66	0.127
**Diastolic blood pressure (mmHg) ($\bar{x}$ ± ds)**	76.23 ± 8.65	74.71 ± 8.9	0.001
**Hypercholesterolemia *n* (%)**	2237 (28.6%)	128 (29.9%)	0.583
**Heart rate (bpm)**	75.70 ± 11.45	72.44 ± 10.77	0.001
**HbA1c ^e^*n* (%)**	7.09 ± 1.26	6.95 ± 1.07	0.035
**Myocardial infarction *n* (%)**	185 (2.4%)	6 (1.4%)	0.247
**Peripheral vascular disease *n* (%)**	323 (4.1%)	29 (6.8%)	0.013
**Valvular disease *n* (%)**	232 (3%)	22 (5.1%)	0.02
**Heart failure *n* (%)**	186 (2.4%)	9 (2.1%)	0.87
**Thromboembolism *n* (%)**	73 (0.9%)	1 (0.2%)	0.185
**CHA_2_DS_2_VASc ^f^ ($\bar{x}$ ± ds)**	4.03 ± 0.99	4.26 ± 0.83	0.005
**Chronic renal insufficiency *n* (%)**	249 (3.2%)	20 (4.7%)	0.094
**Dementia *n* (%)**	203 (2.6%)	7 (1.6%)	0.278
**Insulin *n* (%)**	1221 (15.6%)	76 (17.8%)	0.246
**Oral antidiabetic’s *n* (%)**	5164 (66.1%)	338 (79%)	<0.001
**Beta-blockers *n* (%)**	1383 (17.7%)	112 (26.2%)	<0.001
**Calcium antagonist’s *n* (%)**	1511 (19.3%)	124 (29%)	<0.001
**ACE inhibitors-ARBs ^g^ (%)**	5188 (66.4%)	353 (82.5%)	<0.001
**Diuretics *n* (%)**	1961 (25.1%)	154 (36%)	<0.001

^a^ Atrial fibrillation. ^b^ Student–Fisher *t*-test for continuous variables and Chi square contrast for categorical variables. ^c^ Standard deviation. ^d^ Body mass index ^e^ Glycated hemoglobin. ^f^ Score for estimating the risk of stroke. ^g^ Angiotensin-converting enzyme inhibitors-angiotensin receptor blockers.

**Table 2:** AF incidence density according CHA_2_DS₂VASc score by gender.

CHA₂DS₂VASc	Number of Patients	Number of AF	ID ^a^ Total (95% Confidence Interval, CI)	ID Men	ID Women	*p* ^b^
**0**	0	0	0	0	0	
**1**	0	8	0	0	0	
**2**	693	6	2.31(1.0–4.26)	2.31	0	
**3**	1533	62	8.21 (6.30–10.53)	10.8	1.05	<0.001
**4**	2988	183	12.77 (10.99–14.77)	16.09	4.7	<0.001
**5**	2792	161	12.18 (10.37–14.21)	113.9	1.56	<0.001
**6**	221	12	11.7 (6.07–20.53)	0	11.7	
**7**	10	2	40 (4.84–144.50)	0	40	
**8**	0	0	0	0	0	
**9**	0	0	0	0	0	

^a^ Incidence density. ^b^ Value of statistical significance.

**Table 3:** Independent prognostic factors related to AF incidence at 5 years.

	HR ^a^	95% CI ^b^	*p* ^c^
**Gender**			
**Men**	1		
**Women**	0.55	0.37–0.82	0.004
**Age**	1.07	1.05–1.09	<0.001
**Weight**	1.03	1.02–1.04	<0.001
**Heart rate**	0.98	0.97–0.99	<0.001
**CHA₂DS₂VASc**	1.57	1.16–2.13	0.003

^a^ Hazard ratio. ^b^ 95% confidence interval. ^c^ Statistical Significance Value.

**Table 4:** Atrial fibrillation and stroke-incidence density according to risk quartile and gender.

ID ^a^ /1000/People/Years	Q1	Q2	Q3	Q4	All
**MEN**					
Atrial Fibrillation (95% CI)	3.8 (2.1–6.2)	12.4 (8.9–16.7)	24.4 (18.6–31.4)	37.6 (25.0–54.4)	13.7 (11.6–16.1)
NNS ^b^	53	16	9	6	15
Stroke (95% CI)	0.3 (0.0–1.4)	0.9 (0.2–2.6)	1.6 (0.4–4.2)	8.1 (3.0–17.6)	1.3 (0.7–2.2)
**WOMEN**					
Atrial Fibrillation (95% CI)	0.7 (0.0–3.9)	3.3 (1.3–6.7)	7.4 (4.5–11.5)	19.7 (15.6–24.5)	10.4 (8.6–12.6)
NNS ^b^	284	60	27	10	20
Stroke (95% CI)	-	0.5 (0.0–2.6)	0.4 (0.0–2.1)	2.7 (1.4–4.8)	1.3 (0.7–2.2)
**IQR ^c^ limits**	≤6.88	≤7.71	≤8.39	>8.39	
Atrial Fibrillation (95% CI)	3.0 (1.7–4.5)	8.9 (6.5–11.7)	15.5 (12.3–19.3)	22.5 (18.4–27.1)	12.1 (10.7–13.7)
NNS ^b^	67	22	13	9	17
Stroke (95% CI)	0.2 (0.0–1.0)	0.7 (0.2–1.9)	1.0 (0.3–2.3)	3.5 (2.1–5.7)	1.3 (0.9–1.9)

^a^ Incidence density. ^b^ NNS number-needed-to-screen for one AF case. ^c^ IQR interquartile range.

## Supplementary Materials

The following are available online at https://www.mdpi.com/1660-4601/17/10/3491/s1. Figure S1: Current territory study map, Table S1: Continuous basal variables, Table S2: Categorical basal variables, Table S3. Multivariable-Adjusted by Sex and Interaction for Atrial Fibrillation Risk Factors in the Overall Sample.

## References

[1] King’s College London for the Stroke Alliance for Europe Atrial Fibrillation. https://strokeeurope.eu/.

[2] Clua-Espuny J.L., Lechuga-Durán Í., Bosch R., Roso-Llorach A., Panisello-Tafalla A., Lucas-Noll J., López-Pablo C., Queralt-Tomas L., Giménez-Garcia E., Gonzalez-Rojas N. (2013). Prevalencia de la fibrilación auricular desconocida y la no tratada con anticoagulantes. Estudio AFABE. Rev. Española de Cardiol..

[3] Alkhouli M., Alqahtani F., Aljohani S., Alvi M., Holmes D.R. (2018). Burden of Atrial Fibrillation–Associated Ischemic Stroke in the United States. JACC: Clin. Electrophysiol..

[4] Wang A., Green J.B., Halperin J.L., Piccini J.P. (2019). Atrial Fibrillation and Diabetes Mellitus: JACC Review Topic of the Week. J. Am. Coll. Cardiol..

[5] Echouffo-Tcheugui J.B., Shrader P., Thomas L., Gersh B.J., Kowey P.R., Mahaffey K.W., Singer D.E., Hylek E.M., Go A.S., Peterson E.D. (2017). Care Patterns and Outcomes in Atrial Fibrillation Patients With and Without Diabetes. J. Am. Coll. Cardiol..

[6] Welton N.J., McAleenan A., Thom H., Davies P., Hollingworth W., Higgins J.P.T., Okoli G., Sterne J., Feder G., Eaton D. (2017). Screening strategies for atrial fibrillation: A systematic review and cost-effectiveness analysis. Heal. Technol. Assess..

[7] Zethelius B., Gudbjörnsdottir S., Eliasson B., Eeg-Olofsson K., Svensson A.-M., Cederholm J. (2015). Risk factors for atrial fibrillation in type 2 diabetes: Report from the Swedish National Diabetes Register (NDR). Diabetologia.

[8] Staerk L., Preis S.R., Lin H., Casas J.P., Lunetta K., Weng L.-C., Anderson C.D., Ellinor P.T., Lubitz S.A., Benjamin E.J. (2020). Novel Risk Modeling Approach of Atrial Fibrillation With Restricted Mean Survival Times: Application in the Framingham Heart Study Community-Based Cohort. Circ. Cardiovasc. Qual. Outcomes.

[9] Meyre P., Aeschbacher S., Blum S., Coslovsky M., Beer J.H., Moschovitis G., Rodondi N., Baretella O., Kobza R., Sticherling C. (2020). The Admit-AF risk score: A clinical risk score for predicting hospital admissions in patients with atrial fibrillation. Eur. J. Prev. Cardiol..

[10] Rossello X., Dorresteijn J.A., Janssen A., Lambrinou E., Scherrenberg M., Bonnefoy-Cudraz E., Cobain M., Piepoli M.F., Visseren F.L., Dendale P. (2019). Risk prediction tools in cardiovascular disease prevention: A report from the ESC Prevention of CVD Programme led by the European Association of Preventive Cardiology (EAPC) in collaboration with the Acute Cardiovascular Care Association (ACCA) and the Association of Cardiovascular Nursing and Allied Professions (ACNAP). Eur. J. Prev. Cardiol..

[11] Yang P., Zhao Y., Wong N.D. (2020). Development of a Risk Score for Atrial Fibrillation in Adults With Diabetes Mellitus (from the ACCORD Study). Am. J. Cardiol..

[12] Saliba W., Gronich N., Barnett-Griness O., Rennert G. (2016). Usefulness of CHADS2 and CHA2DS2-VASc Scores in the Prediction of New-Onset Atrial Fibrillation: A Population-Based Study. Am. J. Med..

[13] Ictus: Action Plan in Europe (2018–2030) Ed Stroke Alliance For Europe (SAFE). https://eso-stroke.org/action-plan-stroke-europe-2018-2030-2/.

[14] Moran P.S., Teljeur C., Ryan M., Smith S.M. (2016). Systematic screening for the detection of atrial fibrillation. Cochrane Database Syst. Rev..

[15] Clua-Espuny J.L., Muñoz-Perez M.A., Bustamante-Rangel A. Stepwise High Risk Individuals Screening for Atrial Fibrillation Using Sequential Clinical-electro-biological Register: The AFRICAT Study (Atrial Fibrillation Research In CATalonia).

[16] Hess P.L., Healey J., Granger C.B., Connolly S., Ziegler P.D., Alexander J.H., Kowey P.R., Ruff C., Flaker G., Halperin J.L. (2017). The Role of Cardiovascular Implantable Electronic Devices in the Detection and Treatment of Subclinical Atrial Fibrillation. JAMA Cardiol..

[17] Attia Z.I., Noseworthy P.A., Lopez-Jimenez F., Asirvatham S.J., Deshmukh A.J., Gersh B.J., Carter R.E., Yao X., Rabinstein A.A., Erickson B.J. (2019). An artificial intelligence-enabled ECG algorithm for the identification of patients with atrial fibrillation during sinus rhythm: A retrospective analysis of outcome prediction. Lancet.

[18] Freedman B., Camm J., Calkins H., Healey J.S., Rosenqvist M., Wang J., Albert C.M., Anderson C.S., Antoniou S., Benjamin E.J. (2017). Screening for Atrial Fibrillation. Circulation.

[19] Ballesta-Ors J., Clua-Espuny J.L., Gentille-Lorente D.I., Lechuga-Duran I., Fernández-Saez J., Muria-Subirats E., Blasco-Mulet M., Lorman-Carbo B., Alegret J.M. (2020). Results, barriers and enablers in atrial fibrillation case finding: Barriers in opportunistic atrial fibrillation case finding-a cross-sectional study. Fam. Pr..

[20] Reinke F., Bettin M., Ross L.S., Kochhäuser S., Kleffner I., Ritter M., Minnerup J., Dechering D., Eckardt L., Dittrich R. (2018). Refinement of detecting atrial fibrillation in stroke patients: Results from the TRACK-AF Study. Eur. J. Neurol..

[21] Chan N.-Y. (2018). Systematic Screening for Atrial Fibrillation in the Community: Evidence and Obstacles. Arrhythmia Electrophysiol. Rev..

[22] Lowres N., Olivier J., Chao T.-F., Chen S.-A., Chen Y., Diederichsen A., Fitzmaurice D.A., Gomez-Doblas J.J., Harbison J., Healey J.S. (2019). Estimated stroke risk, yield, and number needed to screen for atrial fibrillation detected through single time screening: A multicountry patient-level meta-analysis of 141,220 screened individuals. PLoS Med..

[23] Lip G.Y., Banerjee A., Boriani G., Chiang C.-E., Fargo R., Freedman B., Lane D.A., Ruff C., Turakhia M., Werring D. (2018). Antithrombotic Therapy for Atrial Fibrillation. Chest.

[24] Vermond R.A., Geelhoed B., Verweij N., Tieleman R.G., Van Der Harst P., Hillege H.L., Van Gilst W.H., Van Gelder I.C., Rienstra M. (2015). Incidence of Atrial Fibrillation and Relationship With Cardiovascular Events, Heart Failure, and Mortality. J. Am. Coll. Cardiol..

[25] Einarson T.R., Acs A., Ludwig C., Panton U.H. (2018). Prevalence of cardiovascular disease in type 2 diabetes: A systematic literature review of scientific evidence from across the world in 2007–2017. Cardiovasc. Diabetol..

[26] Bernet W., Gregory N., Öngider, Reay K.M., Rohner R.P. (2017). An Objective Measure of Splitting in Parental Alienation: The Parental Acceptance-Rejection Questionnaire. J. Forensic Sci..

[27] Pallisgaard J.L., Schjerning A.-M., Lindhardt T.B., Procida K., Hansen M.L., Torp-Pedersen C., Gislason G. (2015). Risk of atrial fibrillation in diabetes mellitus: A nationwide cohort study. Eur. J. Prev. Cardiol..

[28] Tadic M., Cuspidi C. (2015). Type 2 diabetes mellitus and atrial fibrillation: From mechanisms to clinical practice. Arch. Cardiovasc. Dis..

[29] Docherty K.F., Shen L., Castagno D., Petrie M.C., Abraham W.T., Böhm M., Desai A.S., Dickstein K., Køber L.V., Packer M. (2020). Relationship between heart rate and outcomes in patients in sinus rhythm or atrial fibrillation with heart failure and reduced ejection fraction. Eur. J. Hear. Fail..

[30] Holmqvist F., Kim S., Steinberg B.A., Reiffel J.A., Mahaffey K.W., Gersh B.J., Fonarow G.C., Naccarelli G.V., Chang P., Freeman J.V. (2015). Heart rate is associated with progression of atrial fibrillation, independent of rhythm. Heart.

[31] Rosenberg M.A., Gottdiener J.S., Heckbert S.R., Mukamal K.J. (2011). Echocardiographic diastolic parameters and risk of atrial fibrillation: The Cardiovascular Health Study. Eur. Hear. J..

[32] Wang T.J., Parise H., Levy D., D’Agostino R.B., Wolf P., Vasan R.S., Benjamin E.J. (2004). Obesity and the Risk of New-Onset Atrial Fibrillation. JAMA.

[33] Nalliah C.J., Sanders P., Kottkamp H., Kalman J.M. (2015). The role of obesity in atrial fibrillation. Eur. Hear. J..

[34] Magnussen C., Niiranen T., Ojeda F.M., Gianfagna F., Blankenberg S., Njølstad I., Vartiainen E., Sans S., Pasterkamp G., Hughes M. (2017). Sex Differences and Similarities in Atrial Fibrillation Epidemiology, Risk Factors, and Mortality in Community Cohorts: Results From the BiomarCaRE Consortium (Biomarker for Cardiovascular Risk Assessment in Europe). Circulation.

[35] Wu V.C.-C., Wu M., Aboyans V., Chang S.-H., Chen S.-W., Chen M.-C., Wang C.-L., Hsieh I.-C., Chu P.-H., Lin Y.-S. (2019). Female sex as a risk factor for ischaemic stroke varies with age in patients with atrial fibrillation. Heart.

[36] Kaplan R.M., Koehler J., Ziegler P.D., Sarkar S., Zweibel S., Passman R.S. (2019). Stroke Risk as a Function of Atrial Fibrillation Duration and CHA 2 DS 2 -VASc Score. Circulation.

[37] Morillas P., Pallares V., Rubio L.F., Llisterri J.L., Sebastián M.E., Gómez M., Castilla E., Camarasa R., Sandín M., García-Honrubia A. (2015). La puntuación CHADS2 como predictor de riesgo de ictus en ausencia de fibrilación auricular en pacientes hipertensos de 65 o más años. Rev. Española de Cardiol..

[38] Linker D.T., Murphy T.B., Mokdad A.H. (2018). Selective screening for atrial fibrillation using multivariable risk models. Heart.

[39] Wilke T., Groth A., Müller S., Pfannkuche M., Verheyen F., Linder R., Maywald U., Bauersachs R., Breithardt G. (2012). Incidence and prevalence of atrial fibrillation: An analysis based on 8.3 million patients. Europace..

[40] Zungsontiporn N., Link M.S. (2018). Newer technologies for detection of atrial fibrillation. BMJ.

