# Supplementary material for: Incidence and Risk Assessment for Atrial Fibrillation at 5 Years: Hypertensive Diabetic Cohort
Source: Int J Environ Res Public Health. 2020 May 16;17(10):3491. doi: 10.3390/ijerph17103491 (PMC7277633; doi:10.3390/ijerph17103491)
Supplement: Supplementary file 1 [file ijerph-17-03491-s001.pdf]

International Journal of
Environmental Research and Public Health

Supplementary materials

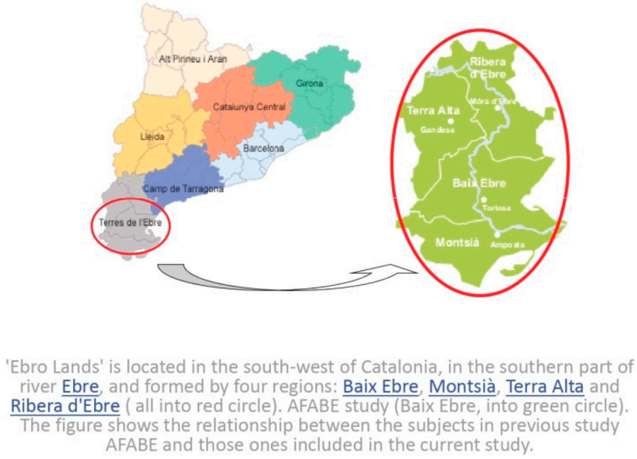

Figure S1. Current territory study map.

Table S1. Continuous basal variables.

Continuous variables	Units	N	Variable modeling
**Following-time**	years	8,237	Average following-time years/patient from 01/01/2013 to patient's death or until the end of the study (31/12/2017).
**Age**	years	8,237	Age (years-old) at the inclusion
**Weight**	kg	4,708	Weight measures average in following time
**BMI**[a]	kg/m^2	3,504	BMI measures average in following time
**Systolic blood pressure (SBP)**	mmHg	6,309	SBP measures average in following time
**Diastolic blood pressure (DBP)**	mmHg	6,253	DBP measures average in following time
**Heart rate (HR)**	bpm	5,501	Heart rate measures average in following time
**Glycated haemoglobin (HbA1c)**	%	5,793	HbA1c measures average in following time
**CHA_2DS_2VASc**		8,237	CHA_2DS_2VASc measures average in following time

[a] Body mass index.

International Journal of
Environmental Research
and Public Health

MDPI

Table S2. Categorical basal variables.

	Units	Variable modeling
**Gender**		**Registered condition in the medical history**
**Diabetes mellitus 2**	mg/dL	Basal glycemia > 126 mg/dL twice; glycemia at 2 hours of the TTOG > 200 mg/dL twice; random glycemia > 200 mg/dL and typical symptoms; HbA1c > 6.5% twice.
**Insulin**		Registered condition in the electronic prescription
**Oral antidiabetics**		Registered condition in the electronic prescription
**Hypertension**	mmHg	Blood pressure > 140/90mmHg (three consecutive measures).
**Beta-blockers**		Registered condition in the electronic prescription
**Calcium antagonists**		Registered condition in the electronic prescription
**ACE inhibitors- ARBs[a]**		Registered condition in the electronic prescription
**Diuretics**		Registered condition in the electronic prescription
**Hypercholesterolemia**	mg/dL	One total cholesterol > 250mg/dL
**Myocardial infarction**		Registered condition in the ICD-10
**Peripheral vascular disease**		Registered condition in the ICD-10
**Valvular disease**		Registered condition in the ICD-10
**Heart failure**		Registered condition in the ICD-10
**Thromboembolism**		Registered condition in the ICD-10
**Chronic renal insufficiency**	ml/min/1.73 m^2	Glomerular filtration < 60 ml/min/1.73 m^2
**Dementia**		Registered condition in the ICD-10

International Journal of
Environmental Research and Public Health

MDPI

Table S3. Multivariable-Adjusted by Sex and Interaction for Atrial Fibrillation Risk Factors in the Overall Sample.

Variable	Interaction	Sex Hazard Ratios		P Value	Relative Risk Ratio (CI95%) (Women/Men)
****Age (years) ($\bar{x}$±ds)**	< 0.001	Women	1.06 (1.01–1.03)	0.001	0.98 (0.89–1.07)
		Men	1.08 (1.04–1.12)	0.01	
****Weight (kg) ($\bar{x}$±ds)**	< 0.001	Women	1.01 (0.98–1.03)	0.372	0.99 (0.96–1.02)
		Men	1.02 (1.01–1.04)	0.001	
**BMI[d](kg/m^2) ($\bar{x}$±ds)**	0.522	Women	1.04 (0.97–1.10)	0.276	1.01 (0.95–1.07)
		Men	1.02 (0.97–1.06)	0.611	
****Diastolic blood pressure (mmHg) ($\bar{x}$±ds)**	0.496	Women	0.99 (0.96–1.03)	0.85	0.95 (0.90–1.0)
		Men	1.04 (0.99–1.04)	0.286	
****Heart rate (bpm)**	< 0.001	Women	0.89 (0.69–1.15)	0.191	0.89 (0.64–1.14)
		Men	0.99 (0.97–1.01)	0.444	
**HbA1c[e] n (%)**	0.496	Women	0.89 (0.69–1.15)	0.374	0.96 (0.54–1.02)
		Men	0.92 (0.77–1.11)	0.392	
**Peripheral vascular disease n (%)**	0.24	Women	1.62 (0.86–48.2.64)	0.152	1.04 (0.79–1.25)
		Men	1.55 (1.08–2.11)	0.075	
**Valvular disease n (%)**	0.24	Women	0.61 (0.22–1.72)	0.35	0.83 (0.0–2.48)
		Men	0.73 (0.26–2)	0.539	
****CHA_2DS_2VASc ($\bar{x}$±ds)**	0.003	Women	3.45 (1.56–7.63)	0.002	2.97 (0.0–6.66)
		Men	1.16 (0.67–1.99)	0.589	
****Oral antidiabetics n (%)**	0.35	Women	0.96 (0.52–1.76)	0.893	1.35 (0.29–2.41)
		Men	0.71 (0.40–1.28)	0.258	
****Beta-blockers n (%)**	0.349	Women	0.88 (0.50–1.55)	0.644	1.14 (0.3–2.01)
		Men	0.77 (0.49–1.19)	0.232	
****Calcium antagonists n (%)**	0.803	Women	0.87 (0.51–1.48)	0.614	0.67 (0.0–1.73)
		Men	1.28 (0.83–2.0)	0.266	
****ACE inhibitors- ARBs[f] (%)**	0.264	Women	1.40 (0.78–2.49)	0.256	3.04 (1.87–4.21)

International Journal of
Environmental Research and Public Health

		Men	0.46 (0.24–0.89)	0.02	
****Diuretics n (%)**	0.609	Women	1.31 (0.79–2.19)	0.293	1.77 (0.77–2.77)
		Men	0.74 (0.49–1.10)	0.137	

[a] Angiotensin converting enzyme inhibitors-angiotensin receptor blockers.
